# A Rapid Method for Performing a Multivariate Optimization of Phage Production Using the RCCD Approach

**DOI:** 10.3390/pathogens10091100

**Published:** 2021-08-29

**Authors:** Jessica Silva, Roberto Dias, José Ivo Junior, Maraísa Marcelino, Mirelly Silva, Adriele Carmo, Maira Sousa, Cynthia Silva, Sergio de Paula

**Affiliations:** 1 Laboratory of Molecular Immunovirology, Department of General Biology, Federal University of Viçosa, Viçosa, Minas Gerais 36570-900, Brazil; jessica.duart.s@gmail.com (J.S.); rosousa318@gmail.com (R.D.); maraisa.marcelino@ufv.br (M.M.); mirellyjady@yahoo.com.br (M.S.); adriele.docarmo27@gmail.com (A.C.); mpsousabio@gmail.com (M.S.); 2 Department of Statistics, Federal University of Viçosa, Viçosa, Minas Gerais 36570-900, Brazil; jivo@ufv.br; 3 Leopoldo Américo Miguez de Mello Research Center (CENPES), Petrobras, Rio de Janeiro 20230-010, Brazil; 4 Department of Microbiology, Federal University of Viçosa, Viçosa, Minas Gerais 36570-900, Brazil; ccanedosilva@gmail.com

**Keywords:** bacteriophage, bioprocess, optimization, RCCD, T4-like phage

## Abstract

Bacteriophages can be used in various applications, from the classical approach as substitutes for antibiotics (phage therapy) to new biotechnological uses, i.e., as a protein delivery vehicle, a diagnostic tool for specific strains of bacteria (phage typing), or environmental bioremediation. The demand for bacteriophage production increases daily, and studies that improve these production processes are necessary. This study evaluated the production of a T4-like bacteriophage vB_EcoM-UFV09 (an *E. coli*-infecting phage with high potential for reducing environmental biofilms) in seven types of culture media (Luria–Bertani broth and the M9 minimal medium with six different carbon sources) employing four cultivation variables (temperature, incubation time, agitation, and multiplicity of infection). For this purpose, the rotatable central composite design (RCCD) methodology was used, combining and comparing all parameters to determine the ideal conditions for starting to scale up the production process. We used the RCCD to set up the experimental design by combining the cultivation parameters in a specific and systematic way. Despite the high number of conditions evaluated, the results showed that when specific conditions were utilized, viral production was effective even when using a minimal medium, such as M9/glucose, which is less expensive and can significantly reduce costs during large-scale phage production.

## 1. Introduction

Phage therapy applies bacteria-infecting viruses, also called phages, to control bacterial infections [1,2]. Since its discovery in the early 20th century, the potential of phages to treat bacterial diseases was quickly recognized. Several studies involving phage cocktails that successfully treated diseases such as cholera, typhoid, and dysentery were developed worldwide in an era before antibiotics. Unfortunately, even with the development of commercial phage-based products, the still insufficient understanding of viral mechanisms, the misuse of phage cocktails over the years, and lastly, the discovery of penicillin mean that phage therapy has almost completely stopped being used [1,2,3,4]. Recently, due to the increase in multidrug-resistant (MDR) bacterial strains, the need for alternative methodologies for controlling pathogenic bacteria has resurfaced, making phage therapy a good alternative [1,3,4]. Although the application of phages for treatment in humans is still re-emerging, essential projects such as the “center for innovative phage applications and therapeutics”, of the UC San Diego School of Medicine in the United States, the Phagoburn (European Union financed clinical study) and the Eliava Phage Therapy Center in Georgia reinforce the return of the phage therapy as a solid alternative treatment against infections caused by pathogenic bacteria [5,6,7]. However, while the application of phages in humans still seldom happens and their use ends up being largely barred by modern pharmaceutical legislation [8,9], their use in veterinary diseases, such as mastitis, and to tackle agricultural and environmental problems associated with biofilm formation has been the subject of recurrent research, presenting promising results [10,11,12,13]. 

When the cells are in a biofilm arrangement, they become more resistant to the action of biocides since extracellular polymeric substances (EPS) form a physical barrier that protects cells from these agents [14,15,16]. Phage tail enzymes, such as depolymerases and lysins, can cleave and disintegrate polymers that compound the biofilm and present a strong biotechnological potential [17,18,19]. Because of this, although the bacterial lysis caused by phages is the focus of phage therapy, its ability to disrupt biofilm or prevent it from being formed already facilitates a treatment against various problems caused by bacteria. This is important not just in a health context, but also from an industrial and environmental perspective, as biofilms are the main factor responsible for microbially-influenced corrosion (MIC) [20]. In a global study, costs related to corrosion were estimated to be around US$2.5 trillion (3.4% of the world’s gross domestic product); however, using available control practices, these costs could be reduced by 15–35% [21,22]. Of the estimated cost from corrosion, between 20–50% is related to MIC [20], and sulfate-reducing bacteria (SRB) are the main microorganisms responsible for MIC in anoxic environments [23].

Sulfate-reducing bacteria are a heterophyletic bacterial group that uses sulfate as a final electron acceptor during respiration, with hydrogen sulfide (H_2_S) as the main product of this metabolism [24,25]. These organisms form biofilms in tanks and pipelines of oil platforms and refineries and are the leading cause of costs associated with the biogenic corrosion process in oil exploration and processing environments. Their presence also accentuates the acidification of the aqueous environment and increases the concentration of sulfur compounds in oil, a phenomenon known as “souring”, which reduces the oil’s economic value [24,26,27]. Biocides such as THPS 75% and Glutaraldehyde 50% are typically used to control SRB growth. Because of the need for an exorbitant amount of these compounds on oil platforms, as well as the microbial resistance that can be developed and the toxicity related to the permanence of these substances even in treated effluents, the search for an alternative methodology to replace or even decrease the use of biocides in oil-related has become an imperative question [14,28,29]. 

Since the SRB group contains more than 30 genera and 200 species [25,30], the isolation of lytic bacteriophages for each of them requires significant effort. Thus, the use of non-specific bacteriophages that produce enzymes such as depolymerases and lysins could be enough to prevent or disrupt bacterial biofilms [11,18,31,32]. However, one of the main limitations to using bacteriophages for bacterial control in environmental systems is the need for more significant amounts of viral particles, which are not generally obtained on a laboratory scale [33,34]. For example, in oil-related environments, the tanks and pipelines can measure thousands of cubic meters, which would require the use of hundreds of liters of phage cocktails to carry out practical bioremediation. To improve phage production, studies and methodologies aimed at optimizing large-scale production and making it economically viable are essential and represent an increasing demand. Although some studies have investigated important cultivation parameters [35,36,37,38], they usually evaluate each one individually, which is not applicable when a real bioprocess occurs. Under real conditions, all these variables will interact with each other, and synergistic or antagonistic effects can alter the individual influence. 

Viral particle propagation is highly related to and dependent on the host metabolism. In bacteriophages, it is essential to provide satisfactory conditions for both bacterial growth and viral propagation [33,39]. Propagation is a crucial step in phage therapy, and some phages can best be produced in a solid medium [40]; however, this method is not widespread, and phage production is generally performed using a liquid medium [41]. Luria–Bertani (LB) broth is a rich and complex medium used for growing enterobacteria, such as *Escherichia coli*. This medium contains several precursors and amino acids that promote high-speed bacterial growth, with a generation time of 20 minutes [42,43]. Despite these advantages, large-scale viral propagation using the LB medium would lead to high manufacturing costs. Alternatively, another less costly culture medium for bacterial growth could be used [33,36]. The minimal medium M9 is composed primarily of salts, ions, and a carbon source and costs almost thirty times less than the LB medium [44]. Although the carbon source is one of the most critical aspects during phage production, other operational parameters can also strongly influence the phage concentration at the end of the process [33,38]. Several studies evaluate how temperature, incubation time, agitation, and MOI can affect viral production, but most of them were performed with only one variable at a time [38,45,46,47,48,49] and do not consider the influences of these parameters in different medium compositions. To improve phage production in bioreactors, understanding how these variables interact with each other is fundamental for knowing which ones will most affect viral particle production or turn the process economically viable. 

Previous analysis has shown that the phage vB_EcoM-UFV09 (*Myoviridae* family, *Tequatrovirus* genus) effectively decreased sulfate-reducing bacteria biofilm formed in a continuous flow system (data not shown). This study presents a fast method for analyzing several culture conditions, aiming to optimize the achievement of more advantageous start-up conditions for large-scale phage production. The rotatable central composite design (RCCD) is a statistical approach used to combine different variable levels systematically [36,50,51,52]. Here, we used the RCCD to determine the best cultivation conditions, or at the very least, to find any indication as to which parameter combinations may produce the best viral yields in a small volume. Our research aimed to establish a starting point for large-scale production, evaluating only the most favorable conditions, thus reducing the number of steps, costs, and time needed to achieve the complete optimized process. The results showed that some carbon sources are preferable for phage production (and that this has no relation to the number of carbon atoms), that some parameter combinations have an antagonistic behavior, and that the model equations were able to indicate the best culture conditions for phage production, at least on the bench scale.

## 2. Results

### 2.1. Bacterial Growth Kinetics

**Figure 1:**
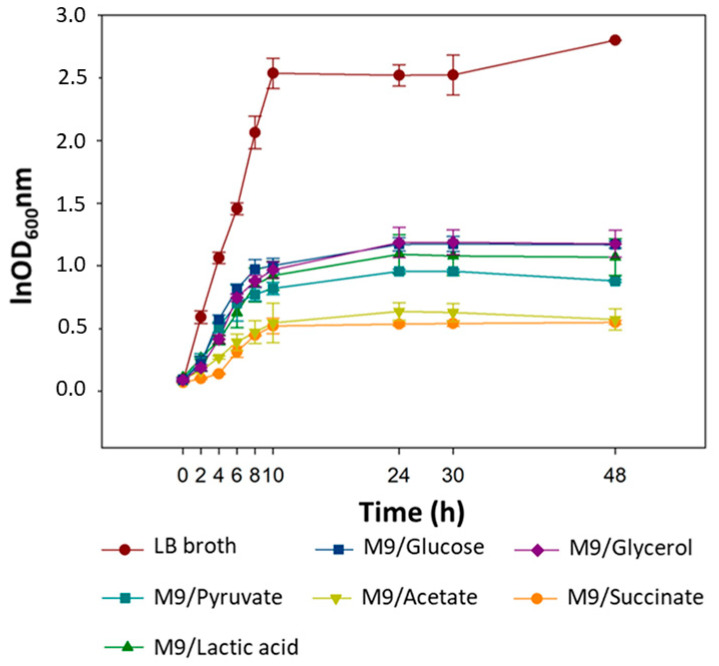
Specific bacterial growth curves for each carbon source are presented. Bacterial growth was monitored for 48 h and DO_600_ was observed at unspecific time intervals. The experiment was performed at 37 °C and 180 rpm agitation.

Bacterial growth in LB medium can be approximately 30 times more expensive than in M9 medium, using glucose or sodium acetate as a carbon source (see Section 4.2). The growth kinetics of *E. coli* 30 in M9 and LB media were evaluated. Based on their growth curves, the natural logarithm (ln) of the slope during the exponential growth phase was used to obtain the intrinsic rate of bacterial growth (µ) in each medium (Figure 1 and Table 1). This analysis allowed the determination of which medium provided the shorter bacterial generation time (g), with more effective growth. The LB medium provided the best growth conditions, with µ being almost four times higher (µ = 1.475) than the best minimal medium evaluated (M9/Glucose, µ = 0.3737), and 8.35 times higher than the poorest minimal medium condition (M9/Acetate, µ = 0.1763). Moreover, the population density in the LB medium (correlated with the OD_600_ values obtained after 24 h of incubation) was more than two times higher than the minimum medium with the highest OD_600_ value (M9/Glucose, OD_600_ = 1.17), and almost five times higher than the minimal medium with the lowest OD_600_ (M9/Succinate, OD_600_ = 0.54). Considering the µ values (Table 1), the patterns of bacterial growth curves, and especially the OD_600_ values, it was possible to observe three distinct behaviors in the overall growth rates (Figure 1). The first one shows the highest growth effectiveness, where the LB medium stands out both for the higher OD_600_ value and for the best µ. The second group demonstrated intermediate effectiveness (M9/Glucose, M9/Glycerol, M9/Lactate, and M9/Pyruvate), and the third showed low bacterial growth (M9/Acetate and M9/Succinate).

### 2.2. Impacts of Operational Cultivation Variables on Bacteriophage Production 

**Figure 2:**
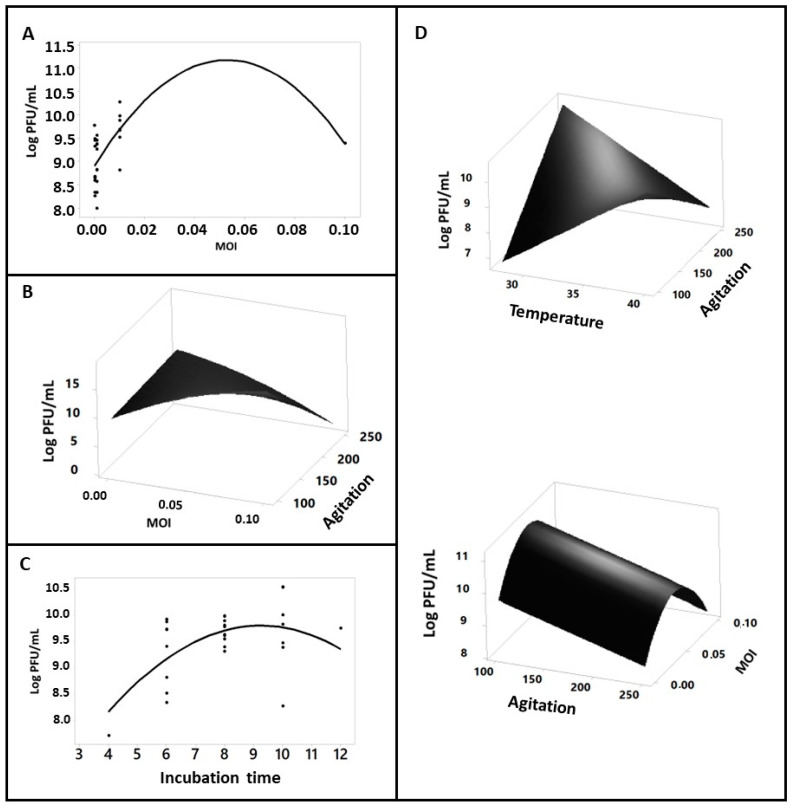
Surface response and dispersion graphics. Equations with just one significant term were plotted in a graphic of dispersion. All surface response graphs resulted from equations for each carbon source. When more than two variables were significant per condition analyzed, the surface response graphs were plotted with two variables included, while others were omitted and fixed at their optimal values, all as functions of the Y axis. (**A**) MOI influenced M9/Glycerol, and the inclination of the curve (parabola form) enabled the acquisition of the maximum value. (**B**) M9/Glucose suffered influence from MOI and agitation. (**C**) LB was only influenced by incubation time. (**D**) M9/Succinate was influenced by three of the variables analyzed, and because of this two surface response graphs were formed.

**Figure 3:**
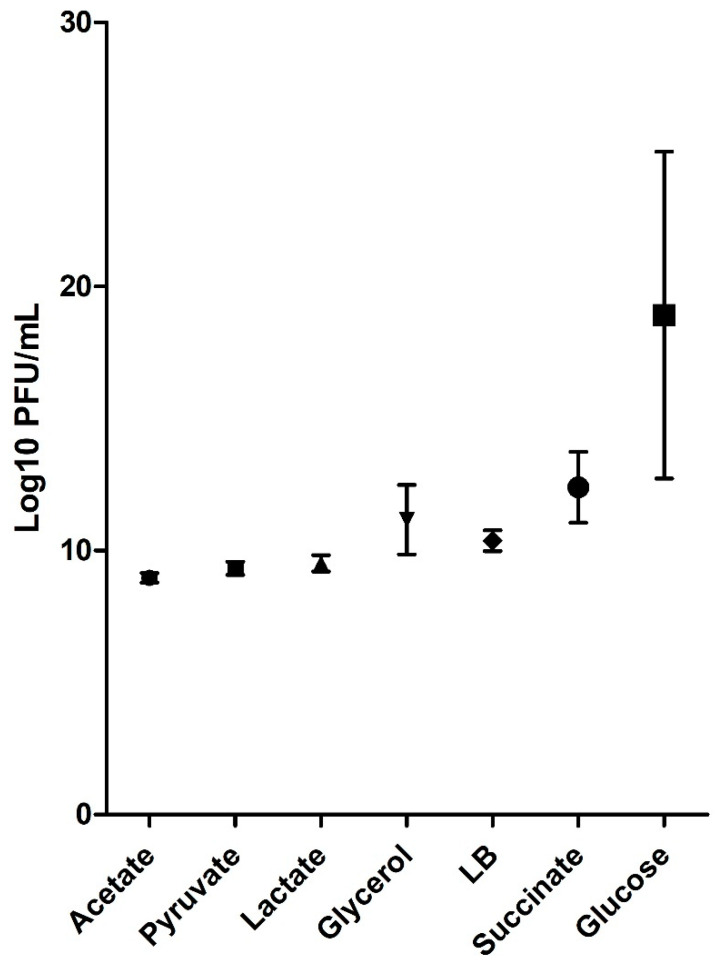
Prediction of the mean values (CI 95%) can be obtained using the best cultivation conditions in each of the media tested. This prediction was based on the correlations presented by the cultivation variables and the determination of their optimal values.

After the 28 assays were performed for each medium individually, it was possible to determine the equation that best synthesizes the influence of the four variables (temperature, incubation time, agitation, and MOI) on the final viral particle production and how these factors are related to each other. The results obtained from the titrations (Table S3) were logarithmized (log_10_). Table 2 presents the regression coefficients obtained by the surface response regression, and Table 3 presents the equations of the quadratic polynomial model predicted by these results. The equations were used to understand the influence of the variables on viral production, statistical analysis, response surface graphs (Figure 2), and production estimates (Figure 3). 

The M9/acetate, M9/Lactate, and M9/Pyruvate media were not influenced significantly (*p* > 0.05) by any variable. Because of this, no response surface graph was obtained for these media (Figure 2).

Cultivation variables that were significant in each experimental unit are summarized in Table 4. MOI was the most significant variable influencing the phage progeny production in three of the seven carbon sources evaluated (3/7; 42.85%), followed by agitation (2/7; 28.57%), which was represented in two carbon sources. Both incubation time and temperature were only significant in one medium: (1/7; 14.28%). 

When a quadratic term is significant in an equation, it is possible to establish the optimal value of this variable, which is reached by solving the derivative equation of this term. This happened in all the media in which the MOI was significant (0.144 PFU/mL in M9/glucose; 0.052 PFU/mL in M9/glycerol, and 0.048 in M9/succinate). If more than one variable is significant in the production process, it is possible to determine how they correlate and influence the final product from the surface response graphs. In the LB medium, the variable that significantly influenced the production was the incubation time (Figure 2C). The resolution of the derivative quadratic term allowed us to predict the ideal incubation time, which was 9 h and 15 min. Phage production in the M9/Glucose minimal medium was influenced by agitation and MOI (Figure 2B). In this medium, agitation and MOI showed a negative interaction with each other: if a reduced agitation was used, a high MOI value was required; on the other hand, if high agitation was used, a lower MOI is needed to achieve reasonable production rates. The optimal MOI number, calculated from the derivative of the quadratic term, was 0.144. 

Unlike the other carbon sources, the M9/Succinate medium was influenced by three variables (Figure 2D). From the linear regression equation, it is possible to infer that temperature presented an antagonistic effect on agitation, and the best results were obtained with the combination of low agitation and high temperature. The optimal calculated MOI was 0.0482. The positive value of the incubation time indicates that more extended experiments produced more phage particles; however, the absence of quadratic terms makes it impossible to determine the optimal production time for the other variables.

### 2.3. Prediction of Viral Particle Production in Different Culture Media

Using the best cultivation conditions obtained by the adjusted equations of the experimental units, the production potential of each medium was predicted (Figure 3). Lower values of the coefficient of determination (R^2^) represent a minor influence for this variable on phage production and a less accurate data prediction. In M9/Acetate, M9/Pyruvate, and M9/Lactate, the prediction was not possible since no culture variable was significant. Therefore, the values shown are based exclusively on the observed experimental phage production. The media M9/Glycerol, M9/Succinate, and LB showed a similar predicted potential of viral production, but the M9/Glucose medium had the best production values. 

### 2.4. Validation of The Mathematical Model 

**Figure 4:**
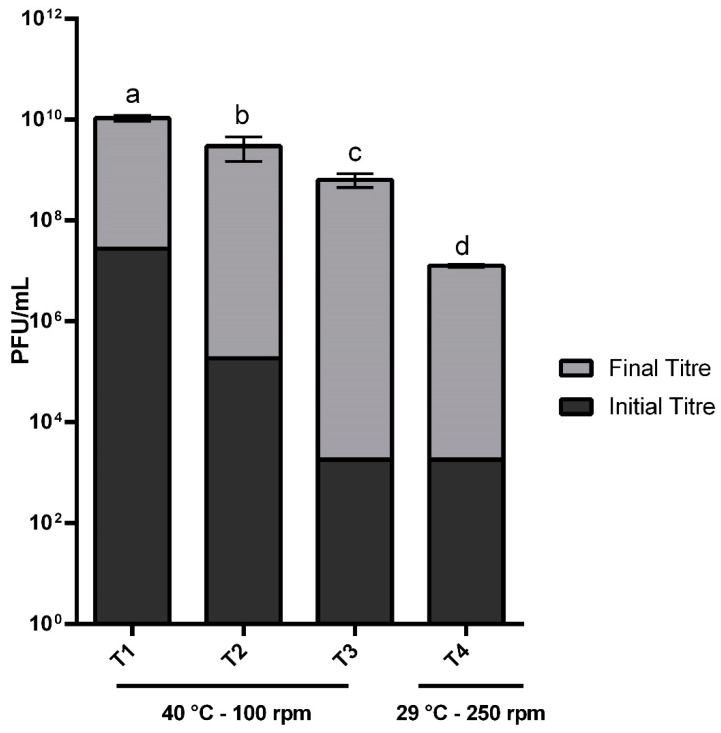
The number of phages recovered after four hours of incubation using different cultivation settings (temperature, agitation and MOI) in M9/Glucose minimal media. We found significant statistical differences between treatments (ANOVA: df = 3, F = 84.45 *p* < 0.001), which are highlighted with letters based on the post-hoc Fisher test (i.e., different letters mean significant differences between treatments). The bars represent the cultivation parameters of each treatment. Legend: T1 = Treatment 1, the best cultivation conditions predicted by the model (40 °C, 100 rpm, and MOI = 0.144); T2 and T3 = Treatments 2 and 3, the same cultivation parameters as T1, but with the intermediate and minimum MOI values tested in this study (MOI = 0.001 and MOI = 0.00001), respectively. T4 = Treatment 4, the worst conditions predicted by the model (29 °C, 250 rpm, and MOI = 0.00001). All the experiments were conducted using an incubation time of four hours (the shortest incubation time tested during this study).

The first assay (Treatment 1) used the best conditions predicted by the model and was the condition that showed the highest yield in absolute numbers, about 1.1 × 10^10^ (Figure 4). Treatment 2 produced fewer phages than the first treatment, with a final mean titer of 3 × 10^9^ PFU/mL, and Treatment 3 produced the smallest number of phages, approximately 6.4 × 10^8^ PFU/mL. While in Treatment 1, the final number of phages recovered was about 405 times higher (~2.5 logs) than the number of inoculating phages, in Treatment 2 this amount was 16,200 times higher (~4 logs), and in Treatment 3 more than 400,000 times higher (~5 logs). Although the lower MOI produced a smaller viral number, it was in this assay that the most significant difference between the number of recovered and inoculated phages was identified, and consequently, where the best proportional yield occurred. It is essential to highlight that with an extra 1.5 h of incubation, the phage number produced using Treatment 3’s conditions became statistically equal to that observed in Treatment 1. Finally, to prove that the RCCD approach’s modeling is reliable and can predict, or at least indicate, good conditions for a bioprocess, we performed the assay with the worst conditions predicted. These cultivation conditions had the same MOI as Treatment 3, but with different temperatures and agitation. The results (Figure 4) showed that these modifications were sufficient to decrease the phage production significantly, since in Treatment 4 the number of phages recovered was 1.26 × 10^7^, a number 51 times smaller than that found in Treatment 3.

## 3. Discussion

Both the OD_600_ values and the intrinsic growth rate of the *E. coli 30* in the LB medium were higher than in the M9 media supplemented with glucose or glycerol (the minimal media with the best growth rates). Our results are similar to those found in other studies that also used complex and minimal media with different carbon sources (including the gluconeogenic ones), and the variations observed in bacterial growth can be explained by differences in the energetic balance, metabolic precursors, and ATP production of each growth medium [53,54]. Some studies correlated medium richness with high phage production and used complex media to perform the experiments [55,56]. However, the use of these media is incompatible with large-scale production, mainly due to elevated production costs. This study showed that even using minimal media, a high phage production can be obtained if the growth conditions (such as temperature, agitation, incubation time, and MOI) are adequately controlled and optimized. For instance, it was observed that carbon sources with fewer carbon atoms did not necessarily lead to insufficient phage production. They behaved in a non-linear form during the trials, showing high and low progeny for different growth conditions. Despite the highly efficient bacterial growth in the LB medium, phage production in this medium was very similar (or even lower) to other minimal media under some cultivation conditions (Table S2). 

The cultivation parameters evaluated in this study affected viral production specifically for each experimental unit (Table 2 and Table 3, Figure 2). This behavior can be explained by some bacterial culture features such as cell size [57], age [58,59,60], life-cycle [61], growth phase [62], and nutrient availability [63,64], which usually influence the phage infection kinetics, altering the latency time and adsorption efficiency, as well as the number of new phage particles produced in a given time (the burst size) [57,60,64,65,66,67,68]. These aspects are directly influenced by environmental conditions like medium composition, temperature, pH, and agitation [36,49,69]. 

Bacterial species have an optimal growth temperature. This key parameter can alter the host physiology and cell membrane structure and affect the availability of phage receptors on the cell surface, decreasing viral infection rates and altering the phage latency time and burst size [36,42,69]. Analyzing the equation (Table 3), it was possible to observe that temperature was significant only in the M9/Succinate (Figure 2D) medium—where the highest amount of virus was recovered in a temperature close to the optimal for the host growth. One possible reason why this term is significant only in one cultivation condition is that the temperature values utilized in this work were within those used for *E. coli* growth [70], and because of this, the bacterial growth remained in a range that did not affect the viral propagation.

Agitation is another crucial aspect of phage infection. In addition to its important role in the aeration dynamics and oxygen availability, it increases the probability of contact between the phage particles and the host cells, leading to an irreversible attachment beginning the infection process [33,39,71]. However, if the agitation is too intense, the phage tails can be broken while the absorption process inhibits the infectious process [72]. The equation obtained from the M9/Glucose (Table 4) showed precisely this interaction between the terms MOI and Agitation, with a negative correlation. A reduced number of phages requires higher agitation to achieve good phage production, probably due to the improved contact between phage and cell host at lower phage concentrations (low MOI). However, when the MOI utilized was high, the results indicated that intense agitation was not necessary. The highest agitation also resulted in decreased phage production in M9/Succinate. Agitation was only significant in M9/Glucose and M9/Succinate (Table 4). The minimal agitation evaluated was enough for infection to be established in all media and, as mentioned before, high agitation reduced the adsorption rate in M9/Glucose and M9/Succinate, probably because of broken phage tails or due to the difficulty of establishing stable adsorption between phage and host [33,72].

Incubation time only influenced the LB medium (Table 3 and Figure 2C). A direct relation was observed, with the increment in incubation time resulting in a corresponding increase in phage production, probably due to a characteristic physiological state of the cells growing in these different carbon sources [69,73]. The nutritional richness of the LB medium provides a high density of host cells, which allows several viral replicative cycles; on the other hand, the complexity of succinate allows for slow but continuous cell growth and more time until a steady state is achieved and phage production is stable throughout the bacterial growth [44,45,74]. 

When a variable had no significant influence on growth conditions, it was assumed that the production would be the same regardless of the value tested. If viral production was similar at the shortest and longest incubation times, it was assumed that there is no need for long incubation periods, thus optimizing production since less time will achieve the exact yield. Incubation time was not significant for most carbon sources, suggesting that when other cultivation variables were used at their best values, the minimum incubation time evaluated (four hours) was sufficient for the phage to establish an equilibrium state with the bacterial host. The phage–host dynamics have been investigated extensively [75,76,77,78,79,80], and, more specifically, the achievement of a stable state between populations may explain the maintenance of the same viral titer from the completion of the first four hours until the end of experiment (12 h) [78,80]. The rapid arrival of the equilibrium state in our study can be explained by the small experimental volume in which the experiments were conducted (5 mL) [33,81,82]. 

The multiplicity of infection (MOI) is probably one of the most critical cultivation variables for phage production in bioreactors. It will determine the dynamics of infection between the virus and host, and consequently, the batch time until the maximum number of viral particles can be obtained [33,39,83]. When a higher number of phage particles was used (MOI of 1 or more), the equilibrium state was reached quickly since it is statistically likely that all cells present in the bioreactor will be infected, and there will not be enough cells left for subsequent rounds. However, if inoculation occurs with a low phage number (MOI of 0.1 or less), the equilibrium state takes longer to achieve, and more infection cycles occur until populations stabilize [33,39]. Due to this behavior, it is necessary to consider the most advantageous production model: a short batching time coupled with a high initial MOI, or a small viral inoculum at the beginning of the process, but with a longer batching time [33,39]. In this study, MOI was significant in the M9 media supplemented with succinate, glycerol, and glucose, considering the incubation times used in this work. 

After all, the significant terms had been identified, and the equations obtained, it was possible to predict the probable viral production in each culture medium using the cultivation parameters indicated by the models (Figure 3). Most of the growth media presented a similar production, except the M9/Glucose, whose production estimate was considerably higher than the others. Although other studies evaluating phage production using minimal medium enriched with glucose have shown promising results, none of them have achieved a production level like that indicated by this prediction [33,38], which was also the case with the experiments performed with this medium in the present work (Table S2). It is important to remember that mathematical predictions do not consider the biological limitations of the organisms involved. Although the model indicates a production potential of 10^25^ PFU/mL in the M9/Glucose medium, we know that this value is biologically and physiologically impossible to achieve.

The M9/Glucose medium was considered the best medium (based on the phage titer obtained experimentally, the production costs, and yield predicted for each carbon source) for the next steps of our scale-up process and was used to validate the generated mathematical model. The experiments conducted using the best conditions predicted by the models did produce the most significant number of viral particles (Treatment 1; Figure 4). The experiments performed under ideal conditions but with intermediate and low MOI values (Treatments 2 and 3; Figure 4) resulted in a proportionally smaller number of viral particles recovered at the end of the experiments, but with a more significant difference between the number of phages harvested and the number of inoculated phages. These results can be explained by the establishing of a steady state between the phage and bacterial populations, or perhaps more time is needed to establish the steady state [33,39]. With an additional 1.5 hours of incubation, the number of phages produced by Treatment 3 became statistically equal to Treatment 1. These results are similar to other studies involving phage production and show that MOI is an essential factor to be considered at the beginning of large-scale phage production since it is this variable that will control the number of infection cycles and the batch time [33,39,83]. The worst conditions predicted by the model were also tested (Treatment 4; Figure 4) and resulted in the lowest viral progeny of all the conditions evaluated. This cultivation procedure had the same MOI as Treatment 3 but with different values for other operational parameters (temperature and agitation). These results show that operational modifications were sufficient to cause a considerable decrease in the phage yield (since the number of recovered phages in Treatment 4 was 51 times smaller than that found in Treatment 3). All these findings demonstrate the efficiency of this method for considering the synergistic and antagonistic effects of the different parameters and for indicating the ideal conditions for starting the optimization of large-scale phage production. 

Although other studies have already used different growing conditions to determine critical parameters for phage production, our study is the most extensive concerning different combinations of carbon sources and cultivation parameters [29,30,31,32,33]. Although pharmaceutical legislation does not contemplate the particularities of using viruses as bacterial control platforms, we believe that the increasingly influential and encouraging perspectives brought about by phage therapy force the recognition of this methodology as a viable and valuable alternative that requires attention [8,9]. In this way, studies investigating the large-scale production of different phages are essential, since their use is relevant not only in clinical, but also in an environmental and industrial context. This requires much more demanding concentrations and production volumes than simple laboratory production.

We therefore consider that studies like ours clarify that a global optimization of phage production is highly unlikely (since it will be dependent on the host, the culture medium, and the phage used) but that mathematical methodologies such as RCCD can help in the fast and robust determination of the parameters that will interfere in the system under study, helping to facilitate the entire production process.

## 4. Materials and Methods 

### 4.1. Bacterial Strain and vB_EcoM-UFV09 Phage

The propagation of the bacteriophage vB_EcoM-UFV9 was performed using the bacterial strain *E. coli 30* isolated from an acute bovine mastitis infection provided by the Brazilian Agricultural Research Corporation (EMBRAPA), which previously proved to be a significant biofilm former [84]. The phage was isolated from water samples taken from the São Bartolomeu River (−20.73094 S and −42.88922 W, using the World Geodetic System WGS84 as datum) in the municipality of Viçosa, Minas Gerais state, Brazil. This isolate is part of the microorganism collection at the Molecular Immunovirology Laboratory (LIVM) at the Federal University of Viçosa (UFV), Viçosa, Minas Gerais state, Brazil. The phage genome was sequenced, and the sequence has been submitted in GenBank under the accession number: 2462144.

### 4.2. M9 Minimal Medium

The M9 minimal medium was prepared according to the protocol described by Sambrook and Russel (2001), with a few modifications (12.8 g of Na_2_HPO_4_.7H2O; 5 g of KH_2_PO_4_; 0.5 g of NaCl; 1 g of NH_4_Cl; 20 mL of MgSO_4_ (0.1 M); 200 μL of CaCl_2_ (0.5 M); 4 g of a carbon source; and distilled water to complete the volume of 1L). Different carbon source concentrations were evaluated, based on the literature (0.2, 0.3 and 0.4%), and 0.4% w/v was chosen since a decrease in bacterial growth in the lower concentrations was observed, probably associated with carbon source limitations. The highest concentration evaluated (0.4%) was not a bottleneck to bacterial growth (the presence of residual carbon source was assessed using an HPLC). LB broth is a widely used commercial culture medium and was used as the control. Seven culture media were evaluated: six distinct forms of the M9 minimal medium, varying the carbon sources (acetate, lactate, pyruvate, glycerol, succinate, and glucose), and the LB broth (Kasvi, São José do Pinhais, PR, Brazil). Table 5 simulates the production costs of the different culture media used in *E. coli* growth.

### 4.3. Bacterial Growth Kinetics

The growth kinetics of *E. coli* 30 in the M9 (with the six different carbon sources) and LB media were compared to evaluate the intrinsic growth rate of this isolate in the different media and carbon sources. An aliquot of grown bacteria (from a single colony, inoculated into the respective media and kept overnight at 37 °C and 150 rpm) was added to a 50-mL tube with 7 mL of sterile medium until the OD_600_ reached 0.1. The experiment was performed at 37 °C and 180 rpm agitation. During the incubation period, 300-μL aliquots of each medium were taken at different times (fixed intervals), using the SP-1105 Spectrophotometer (Shanghai Spectrum Instruments Co., Shanghai, China) to monitor the optical density (OD_600_). 

### 4.4. Rotatable Central Composite Design (RCCD) and the DOE (Design of Experiment) Optimization Process

Different conditions of viral production were evaluated through an RCCD approach. The effects of four variables (*n* = 4) were analyzed: temperature (Te), incubation time (IT), agitation (Ag), and multiplicity of infection (MOI), with five levels each (Table 6). The experimental design was generated using the Minitab software (Minitab, Inc., State College, PA, USA), composed of 16 possible combinations between the factorial points (2^4^), eight axial assays, and four repetitions of the central point, totaling 28 assays. The experimental design was performed using the follow steps in Minitab software: Stats > DOE (Design of experiments) > Surface response > Creating a surface response experiment > Type of experiment (Central composite) > Number of continuous factors (4) > Experiments (Number of Central Points = 4). All others parameters were kept as defaults. The tables of coded and uncoded values used in this work for each experimental unit are detailed in Supplementary Material (Tables S1 and S2). The range of established values for the selected variables was defined based on previous studies [38,45,84]. Each M9 culture medium with a specific carbon source (acetate, glycerol, glucose, lactate, pyruvate, and succinate) and the LB was considered a separate experimental unit. Thus, the 28 assays defined in the RCCD were performed for each of them, totaling 196 assays. No blocking was applied in this study, and the experiments were performed at random. 

The complete second-order model utilized to adjust the phage production optimization was:

$$\hat{Y}={\hat{\beta }}_{0}\;+\;{\hat{\beta }}_{1}X_{1}\;+\;{\hat{\beta }}_{2}X_{2}\;+\;{\hat{\beta }}_{3}X_{3}\;+\;{\hat{\beta }}_{4}X_{4}\;+\;{\hat{\beta }}_{11}X_{1}^{2}\;+\;{\hat{\beta }}_{22}X_{2}^{2}+\;{\hat{\beta }}_{33}X_{3}^{2}\;+\;{\hat{\beta }}_{44}X_{4}^{2}\;+\;{\hat{\beta }}_{12}X_{1}X_{2}\;+\;{\hat{\beta }}_{13}X_{1}X_{3}\;+\;{\hat{\beta }}_{14}X_{1}X_{4}\;+\;{\hat{\beta }}_{23}X_{2}X_{3}\;+\;{\hat{\beta }}_{24}X_{2}X_{4}\;+\;{\hat{\beta }}_{34}X_{3}X_{4}$$

${\hat{\beta }}_{0},\;{\hat{\beta }}_{1\;},\;{\hat{\beta }}_{2},\;{\hat{\beta }}_{3\;}$and ${\hat{\beta }}_{4}$ are regression coefficients estimators and X_1_, X_2_, X_3_ and X_4_ are independent variables.

### 4.5. Assembly of RCCD Experiments

The bacterial inoculum was previously grown in each culture medium until it reached an OD_600_ of 0.7. Then it was added to a 50 mL tube and diluted with the corresponding culture medium until it reached an initial OD_600_ of 0.1 (approximately 3 × 10^8^ colony-forming units—CFU/mL) to a 5 mL final volume. These cultures were then subjected to the different cultivation conditions predicted by the RCCD (Table S1). The quantification of viral particles (expressed as plaque-forming units per mL, PFU/mL) was made at the end of each experiment using a well-established double-agar overlay plaque assay. Briefly, 100 μL of each sample were collected, and serial dilutions were performed at the ratio of 1:10 using SM buffer (5.8 g of NaCl; 2 g of MgSO_4_; 6 g of Tris.HCl; 1 g of powdered gelatin; distilled water to 1 L, with the pH adjusted to 7). A total of 100 μL was withdrawn and mixed with 700 μL of an E. coli 30 culture grown the previous night from each dilution. This suspension was mixed with 5 mL of top agar LB medium (0.7% agar) and then poured into a plate with solid agar (1.4% agar).

### 4.6. Statistical Analysis and Elaboration of Surface Response Graphs 

Linear regression (Response surface regression) was applied to each experimental unit to determine the significant terms (variables). The steps to perform this analysis in Minitab were: Stats > DOE (Design of experiments) > Surface response > Analising an Experiment of Surface Response > Answers (Y = logarithmized (log10) phage titre values (PFU/mL) observed). All others parameters were kept as defaults. The final equation models were obtained, and the analyses were performed. Optimization criteria were set to maximize phage production (PFU/mL). The equations derived from each experimental unit allowed the generation of surface response graphics (Stats > DOE (Design of experiments) > Surface response > Surface response graphs), which summarize the influence of the significant variables about the phage particle number and aid in understanding how these variables correlated during the production process. The R^2^ value represents how the independent variables explain much variation in the response variable (viral production). The closer these values are to 100%, the more the results correspond to the influence of these significant variables present in the equation. The equations make it possible to understand how each variable influenced phage production (i.e., positively or negatively) and if the interactions between them had an antagonistic or synergistic effect. They were used to interpret and produce the surface response graphs.

### 4.7. Prediction of Viral Particle Production in Different Culture Media

The Minitab software (Minitab, Inc., State College, PA, USA) “predict” tool was used to calculate the phage titer that can be mathematically obtained under the different conditions evaluated (Stats > DOE (Design of experiments) > Surface response > Predict). This analysis is based on the equations obtained for each culture medium and uses the best combinations of the culture parameters to predict the amount of phage that can be produced at the end of a batch.

### 4.8. Model Validation

To validate the model predicted and to evaluate the impact of MOI on phage production, some additional experiments were performed, all with four hours of incubation time, and the same culture medium described previously. The mathematical model was validated for the medium that presented the best potential for commercial use based on the variables evaluated. The best cultivation conditions predicted by the model (40 °C, 100 rpm and MOI = 0.144; treatment 1) and the worst (29 °C, 250 rpm and MOI = 0.00001; treatment 4) were reproduced using the incubation time of four hours (the shortest incubation time tested during this study). To evaluate how the number of phage particles inoculated at the beginning of the experiment can alter the steady-state dynamics of phage and host populations, and the impact of this single operational variable on the final production, we also tested the intermediate and minimum MOI values used in this study under the best cultivation variables (MOI = 0.001 and MOI = 0,00001, treatments 2 and 3, respectively).

The viral quantification was made using the double-agar overlay plaque assay. The average number of phage particles obtained from the four experimental conditions was compared using variance (ANOVA) analysis, followed by a Fisher post-hoc test in case of significance.

## 5. Conclusions

The RCCD approach made it possible to evaluate several operational parameters simultaneously, as well as the relationship between them, independent of the phage/host pair. If the complete factorial method had been used, instead of the 196 tests we performed during this study, at least seven thousand tests would have been needed to obtain the same amount of information. 

The RCCD approach is an exciting and quick tool for obtaining the best parameters for a start a large-scale phage production optimization. Agitation was the parameter that most influenced phage production, while incubation time was the least influential parameter. Factors such as economic viability, production reliability, and physiological stability make the M9/Glucose medium the one that provided the best performance in vB_EcoM-UFV09 T4-like phage production. Although we used these four different parameters in this work, this tool can be used to evaluate any other parameter, such as dissolved oxygen or micronutrients.

## Figures and Tables

**Table 1:** OD_600_ values were obtained after 24 h of experiment and the intrinsic growth rate (µ) of *E. coli* 30 bacteria in the different media was evaluated.

Medium	OD_600_ Mean (24 h)	µ
LB	2.52	1.475
M9/Glucose	1.17	0.3737
M9/Glycerol	1.19	0.357
M9/Pyruvate	0.96	0.3485
M9/Succinate	0.54	0.2142
M9/Lactic Acid	1.09	0.1943
M9/Acetate	0.64	0.1763

µ—intrinsic growth rate is determined by the angular coefficient in a growth curve equation during the logarithmic growth phase. LB = Luria Bertani Broth.

**Table 2:** Regression coefficients obtained by the surface response regression.

Coefficients	Acet.	Pyruv.	Gluc.	Glyc.	Succi.	LB
β0	9.247	11.95	11.136	11.191	10.673	10.725
Linear						
β1 (Te)	−0.28	−2.19	−1.64	0.05	−1.04	0.25
β2 (IT)	0.48	−0.55	1.03	−0.00	−0.64	1.93
β3 (Ag)	1.990	−4.88	−4.22 *	−3.37	0.83	−3.33
β4 (MOI)	0.125	−0.091	0.036	0.245	−0.22	−0.022
Quadratic						
β11 (Te^2^)	−0.797	−1.254	−0.039	−0.158	−0.268	−0.129
β22 (IT^2^)	−0.257	−0.691	−0.632	0.142	−0.410	−1.056 *
β33 (Ag^2^)	0.209	−0.392	−0.042	0.040	−0.360	−0.135
β44 (MOI^2^)	−0.293	−1.358	−1.347 *	−2.048 *	−1.675 *	−1.075
Interactions						
β12 (Te*IT)	0.687	0.473	−0.090	0.577	0.173	−0.481
β13 (Te*Ag)	0.050	−1.426	−0.473	0.539	−1.720 *	−0.099
β14 (Te*MOI)	0.30	−2.48	−2.16	0.12	−1.25	−0.35
β23 (IT*Ag)	−0.340	0.098	−0.684	−0.480	0.268	−0.130
β24 (IT*MOI)	0.25	−0.96	1.10	0.00	−1.15	1.44
β34 (Ag*MOI)	2.00	−5.75	−4.52 *	−3.85	0.69	−3.30
R^2^ adj. (%)	21.31	12.60	32.31	3.42	31.29	34.23

Values with * were significant by the F test (*p* < 0.05). R^2^ values indicate how much of the observed results were explained by the independent variables analyzed. The adjusted R^2^ is a modified version of R^2^ that has been fitted for the number of predictors and is considered a best model evaluator index. Abbreviations: Acet. = M9/acetate; Pyruv. = M9/pyruvate; Gluc. = M9/glucose; Gly. = M9/glycerol; Succi. = M9/succinate; Te = Temperature; IT = Incubation Time; Ag = Agitation; MOI = Multiplicity of Infection.

**Table 3:** Equations obtained from linear regression analyses. The carbon sources are ordered according to the number of carbon atoms from the lowest to the highest. X_1_ (Te) = temperature (28 °C ≤ Te ≤ 40 °C); X_2_ (IT) = incubation time (4 h ≤ IT ≤ 12 h); X_3_ (Ag = agitation (100 rpm ≤ Ag ≤ 250 rpm) and X_4_ (MOI) = multiplicity of infection (0,00001 ≤ MOI ≤ 0,1). Numbers showing (*) had values within the significance range by F test (*p* < 0.05).

Medium	Equations
**Acetate**	$9.247-0.28X_{1}+0.48X_{2}+1.990X_{3}+0.25X_{4}-0.797X_{1}^{2}-0.257X_{2}^{2}+0.209X_{3}^{2}\;-0.293X_{4}^{2}+0.687X_{1}X_{2}+0.050X_{1}X_{3\;}+0.3X_{1}X_{4}-0.34X_{2}X_{3}+0.25X_{2}X_{4}\;+2.00X_{3}X_{4}$
**Lactate**	$10.285-0.84X_{1}+0.15X_{2}+1.15X_{3}-0.013X_{4}-0.111X_{1}^{2}+0.082X_{2}^{2}-0.052X_{3}^{2}\;-0.692X_{4}^{2}-0.043X_{1}X_{2}-0.176X_{1}X_{3\;}-0.8X_{1}X_{4}-0.023X_{2}X_{3}+0.253X_{2}X_{4}+1.23X_{3}X_{4}$
**Pyruvate**	$11.95-2.19X_{1}-0.55X_{2}-4.88X_{3}-0.091X_{4}-1.254X_{1}^{2}-0.61X_{2}^{2}-0.392X_{3}^{2}\;-1.358X_{4}^{2}+0.473X_{1}X_{2}-1.426X_{1}X_{3\;}-2.48X_{1}X_{4}+0.098X_{2}X_{3}-0.96X_{2}X_{4}\;-5.75X_{3}X_{4}$
**Glucose ^ab^**	$11.136-1.64X_{1}+1.03X_{2}-4.22^{*}X_{3}+0.036X_{4}-0.039X_{1}^{2}-0.632X_{2}^{2}-0.042X_{3}^{2}\;-1.358^{*}X_{4}^{2}-0.090X_{1}X_{2}-0.473X_{1}X_{3\;}-2.16X_{1}X_{4}-0.684X_{2}X_{3}+1.10X_{2}X_{4}-4.52^{*}X_{3}X_{4}$
**Glycerol ^a^**	$11.191+0.05X_{1}-0.00X_{2}-3.37X_{3}+0.245X_{4}-0.158X_{1}^{2}+0.142X_{2}^{2}+0.040X_{3}^{2}\;-2.048^{*}X_{4}^{2}+0.577X_{1}X_{2}+0.539X_{1}X_{3\;}+0.12X_{1}X_{4}-0.480X_{2}X_{3}+0.00X_{2}X_{4}\;-3.85X_{3}X_{4}$
**Succinate ^ab^**	$10.673-1.04X_{1}-0.64X_{2}+0.83X_{3}-0.22X_{4}-0.268X_{1}^{2}-0.410X_{2}^{2}-0.360X_{3}^{2}\;-1.675^{*}X_{4}^{2}+0.173X_{1}X_{2}-1.720^{*}X_{1}X_{3\;}-1.25X_{1}X_{4}+0.268X_{2}X_{3}-1.15X_{2}X_{4}+0.69X_{3}X_{4}$
**LB ^a^**	$10.725+0.25X_{1}+1.93X_{2}-3.33X_{3}-0.022X_{4}-0.129X_{1}^{2}-1.056^{*}X_{2}^{2}-0.135X_{3}^{2}\;-1.075X_{4}^{2}-0.481X_{1}X_{2}-0.099X_{1}X_{3\;}-0.35X_{1}X_{4}-0.130X_{2}X_{3}+1.44X_{2}X_{4}-3.30X_{3}X_{4}$

^a^ The presence of the quadratic term in the equations allows the establishment of the optimal value of this variable, aiming for the highest production possible. This value is reached by resolving the derivative equation of this term. ^b^ The presence of two variables multiplied by each other in the same term is considered an interaction between these variables (as occurred in M9/Glucose, with the interval “−4.52*X_3_X_4_”). If this interaction has a negative sign, this indicates that the variables involved are antagonistic; thus, production is best when combining the highest value of one and the lowest value of the other, or vice versa. If an interaction has a positive sign, this indicates that the variables involved are synergistic, and the output is best when the highest value or the lowest value of both are combined. When one of the evaluated cultivation variables is not significant for the production model, it means that when using any value within the range of the experiment, the final production will be the same.

**Table 4:** Variables that influenced the production of viral progeny under different culture conditions.

Medium	Temperature (Te)	Incubation Time (It)	Agitation (Ag)	Multiplicity of Infection (MOI)
Acetate	-	-	-	-
Lactate	-	-	-	-
Pyruvate	-	-	-	-
Glycerol	-	-	-	X
Succinate	X	-	X	X
Glucose	-	-	X	X
LB	-	X	-	-

“X” indicates that the variable influenced the response (by F test; *p*-value < 0.05); “-“ indicates that the variable was not significant.

**Table 5:** Carbon sources used and respective prices.

Carbon Source	Price (US$)	Price/Liter * [0.4%] (US$)	Total Price, M9 Medium + Carbon Source
LB Broth, Miller	169.00/Kg	4.23 **	4.23 **
M9 medium without carbon source	0.09/L	0.09	-
Glucose	7.08/Kg	0.027	0.177
Glycerol	9.28/L	0.036	0.186
Lactic Acid	12.57/L	0.048	0.198
Sodium Acetate	9.66/Kg	0.038	0.188
Sodium Pyruvate	400/Kg	1.59	1.74
Sodium Succinate	340.20/Kg	1.36	1.51

(*) Price/liter considering the cost of preparing 1 L of LB and M9 medium and quantities needed to reach 0.4% of each carbon source. (**) It is not possible to determine the carbon source concentrations in the LB medium (complete medium powder), so 25 g/L were used following the manufacturer’s recommendation. All values considered in these calculations were obtained on 29/04/2019 from different companies that supply laboratories.

**Table 6:** Values used in the RCCD to analyze phage particle production for vB_EcoM-UFV09 with different culture variables and culture media.

Variables	Levels				
	−α	−1	0	+1	+α
Temperature (°C/X_1_)	28	31	34	37	40
Incubation Time (h/X_2_)	4	6	8	10	12
Agitation (rpm/X_3_)	100	138	175	213	250
MOI (PFU/mL/X_4_)	0.00001	0.0001	0.001	0.01	0.1

## Data Availability

Not applicable.

## Supplementary Materials

The following are available online at https://www.mdpi.com/article/10.3390/pathogens10091100/s1, Table S1: Experimental design used by each experimental unit (LB medium and M9 media supplemented with acetate, pyruvate, lactate, glycerol, succinate, and glucose), based on the rotatable central composite design. The variables were: Te = temperature (28 °C ≤ Te ≤ 40 °C); It = incubation time (4 h ≤ It ≤ 12 h); Ag = agitation (100 rpm ≤ Ag ≤ 250 rpm); and MOI = multiplicity of infection (0.00001 ≤ MOI ≤ 0.1). Table S2: Number of phages (PFU/mL) observed at the end of the experiments. Quantification was performed using a double-agar assay. Table S3: Amount of phages (PFU/mL) observed in the end of the experiments. The quantification was made using double-agar assay.
