# Supplementary material for: A Rapid Method for Performing a Multivariate Optimization of Phage Production Using the RCCD Approach
Source: Pathogens. 2021 Aug 29;10(9):1100. doi: 10.3390/pathogens10091100 (PMC8468216; doi:10.3390/pathogens10091100)
Supplement: Supplementary file 1 [file pathogens-10-01100-s001.zip › pathogens-1346604-supplementary.pdf]

Pathogens

Supplementary material

Table S1: Experimental design used by each experimental unit (LB medium and M9 media supplemented with acetate, pyruvate, lactate, glycerol, succinate and glucose), based on Rotatable Central Composite Design. The variables were: Te = temperature (28 °C <= Te <= 40 °C); It = incubation time (4 h <= It <= 12 h); Ag = agitation (100 rpm <= Ag <= 250 rpm) and MOI = Multiplicity of Infection (0.00001 <= MOI <= 0.1).

Assay	Te (°C)	It (h)	Ag (rpm)	MOI
1	31	6	138	0.01
2	37	6	138	0.01
3	31	10	138	0.01
4	37	10	138	0.01
5	31	6	215	0.01
6	37	6	215	0.01
7	31	10	215	0.01
8	37	10	215	0.01
9	31	6	138	0.0001
10	37	6	138	0.0001
11	31	10	138	0.0001
12	37	10	138	0.0001
13	31	6	215	0.0001
14	37	6	215	0.0001
15	31	10	215	0.0001
16	37	10	215	0.0001
17	28	8	175	0.001
18	40	8	175	0.001
19	34	4	175	0.001
20	34	12	175	0.001
21	34	8	100	0.001
22	34	8	250	0.001
23	34	8	175	0.10
24	34	8	175	0.00001
25	34	8	175	0.001
26	34	8	175	0.001
27	34	8	175	0.001
28	34	8	175	0.001

Table S2: Experimental design used by each experimental unit in coded (LB medium and M9 media supplemented with acetate, pyruvate, lactate, glycerol, succinate and glucose), in coded values, based on Rotatable Central Composite Design.

Assay	Te (°C)	It (h)	Ag (rpm)	MOI
1	-1	-1	-1	+1
2	+1	-1	-1	+1
3	-1	+1	-1	+1
4	+1	+1	-1	+1
5	-1	-1	+1	+1
6	+1	-1	+1	+1
7	-1	+1	+1	+1
8	+1	+1	+1	+1
9	-1	-1	-1	-1
10	+1	-1	-1	-1
11	-1	+1	-1	-1
12	+1	+1	-1	-1
13	-1	-1	+1	-1
14	+1	-1	+1	-1
15	-1	+1	+1	-1
16	+1	+1	+1	-1
17	-α	0	0	0
18	+α	0	0	0
19	0	-α	0	0
20	0	+α	0	0
21	0	0	- α	0
22	0	0	+ α	0
23	0	0	0	+ α
24	0	0	0	- α
25	0	0	0	0
26	0	0	0	0
27	0	0	0	0
28	0	0	0	0

Table S3: Amount of phages (PFU/mL) observed in the end of the experiments. The quantification was made using double-agar assay.

Assay	acetate	lactate	pyruvate	glycerol	succinate	glucose	LB
**1**	8.67E+08	5.64E+09	5.53E+09	4.66E+09	7.67E+08	7.86E+09	2.57E+09
**2**	7.44E+07	5.28E+09	4.71E+09	4.90E+09	1.70E+09	9.56E+09	5.42E+09
**3**	4.23E+08	5.73E+09	2.76E+09	1.89E+10	2.80E+09	8.29E+10	3.46E+10

**4**	4.47E+08	6.63E+09	6.74E+09	4.92E+09	6.93E+09	4.42E+10	3.38E+10
**5**	2.74E+09	2.42E+10	6.13E+09	9.52E+09	1.42E+10	1.04E+10	2.21E+08
**6**	1.50E+08	4.28E+09	3.23E+08	3.32E+09	1.33E+09	1.05E+10	7.44E+09
**7**	1.00E+09	7.60E+09	2.91E+09	6.53E+08	3.87E+09	1.41E+09	2.43E+09
**8**	1.64E+09	6.24E+09	7.66E+09	7.59E+09	4.33E+09	6.20E+09	6.77E+09
**9**	5.91E+08	1.09E+10	6.77E+06	4.76E+08	5.83E+07	3.58E+08	3.31E+08
**10**	2.76E+07	6.80E+09	1.28E+09	4.40E+08	5.43E+09	1.38E+10	8.36E+09
**11**	4.02E+08	3.84E+09	2.30E+07	1.83E+08	9.33E+07	4.12E+08	1.91E+08
**12**	4.84E+08	3.58E+09	8.12E+09	3.96E+08	1.97E+09	1.32E+10	1.01E+10
**13**	4.69E+08	2.44E+09	2.14E+09	2.14E+09	7.03E+08	3.51E+09	6.66E+08
**14**	5.82E+08	7.94E+09	3.90E+09	2.83E+09	5.03E+07	8.98E+09	5.38E+09
**15**	1.17E+09	5.82E+09	7.32E+09	2.19E+08	6.13E+09	6.69E+09	2.46E+09
**16**	8.47E+07	1.49E+09	3.87E+09	3.07E+09	7.20E+08	4.74E+09	2.97E+09
**17**	6.11E+08	2.82E+09	2.82E+09	2.84E+09	1.63E+09	4.73E+09	3.51E+09
**18**	4.08E+07	2.78E+09	7.60E+07	2.17E+08	2.00E+09	8.3E+09	6.09E+09
**19**	2.70E+08	3.29E+09	7.87E+08	6.72E+08	3.77E+08	2.24E+09	5.22E+07
**20**	1.11E+09	5.78E+09	3.63E+09	3.64E+09	4.50E+09	1.14E+09	5.71E+09
**21**	2.58E+09	2.71E+09	1.69E+09	6.48E+08	9.27E+08	4.91E+09	9.80E+09
**22**	9.84E+08	3.80E+09	6.44E+09	2.34E+09	2.47E+09	7.8E+09	2.07E+09
**23**	1.20E+09	3.80E+09	3.18E+09	2.44E+09	6.03E+08	6.68E+09	4.26E+09
**24**	2.97E+08	4.40E+09	6.11E+09	5.92E+09	7.43E+08	8.76E+09	9.58E+09
**25**	9.07E+08	1.86E+09	7.11E+09	1.82E+09	4.59E+09	5.09E+09	2.49E+09
**26**	4.70E+09	3.33E+09	6.86E+09	2.4E+09	4.60E+09	5.24E+09	4.16E+09
**27**	6.53E+08	6.34E+09	5.73E+09	3.69E+08	1.63E+09	8.59E+09	7.86E+09
**28**	3.92E+08	5.63E+09	5.96E+09	9.89E+07	2.43E+09	5.49E+09	6.42E+09
